# Preventable Premature Deaths from the Five Leading Causes of Death in Nonmetropolitan and Metropolitan Counties, United States, 2010–2022

**DOI:** 10.15585/mmwr.ss7302a1

**Published:** 2024-05-02

**Authors:** Macarena C. García, Lauren M. Rossen, Kevin Matthews, Gery Guy, Katrina F. Trivers, Cheryll C. Thomas, Linda Schieb, Michael F. Iademarco

**Affiliations:** ^1^Office of Science and Medicine, Office of the Assistant Secretary of Health, U.S. Department of Health and Human Services, Washington, DC; ^2^National Center for Health Statistics, CDC, Hyattsville, Maryland; ^3^National Center for State, Tribal, Local, and Territorial Public Health Infrastructure and Workforce, CDC, Atlanta, Georgia; ^4^National Center for Injury Prevention and Control, CDC, Atlanta, Georgia; ^5^National Center for Chronic Disease Prevention and Health Promotion, CDC, Atlanta, Georgia

## Abstract

**Problem/Condition:**

A 2019 report quantified the higher percentage of potentially excess (preventable) deaths in U.S. nonmetropolitan areas compared with metropolitan areas during 2010–2017. In that report, CDC compared national, regional, and state estimates of preventable premature deaths from the five leading causes of death in nonmetropolitan and metropolitan counties during 2010–2017. This report provides estimates of preventable premature deaths for additional years (2010–2022).

**Period Covered:**

2010–2022.

**Description of System:**

Mortality data for U.S. residents from the National Vital Statistics System were used to calculate preventable premature deaths from the five leading causes of death among persons aged <80 years. CDC’s National Center for Health Statistics urban-rural classification scheme for counties was used to categorize the deaths according to the urban-rural county classification level of the decedent’s county of residence (1: large central metropolitan [most urban], 2: large fringe metropolitan, 3: medium metropolitan, 4: small metropolitan, 5: micropolitan, and 6: noncore [most rural]). Preventable premature deaths were defined as deaths among persons aged <80 years that exceeded the number expected if the death rates for each cause in all states were equivalent to those in the benchmark states (i.e., the three states with the lowest rates). Preventable premature deaths were calculated separately for the six urban-rural county categories nationally, the 10 U.S. Department of Health and Human Services public health regions, and the 50 states and the District of Columbia.

**Results:**

During 2010–2022, the percentage of preventable premature deaths among persons aged <80 years in the United States increased for unintentional injury (e.g., unintentional poisoning including drug overdose, unintentional motor vehicle traffic crash, unintentional drowning, and unintentional fall) and stroke, decreased for cancer and chronic lower respiratory disease (CLRD), and remained stable for heart disease. The percentages of preventable premature deaths from the five leading causes of death were higher in rural counties in all years during 2010–2022. When assessed by the six urban-rural county classifications, percentages of preventable premature deaths in the most rural counties (noncore) were consistently higher than in the most urban counties (large central metropolitan and fringe metropolitan) for the five leading causes of death during the study period.

During 2010–2022, preventable premature deaths from heart disease increased most in noncore (+9.5%) and micropolitan counties (+9.1%) and decreased most in large central metropolitan counties (−10.2%). Preventable premature deaths from cancer decreased in all county categories, with the largest decreases in large central metropolitan and large fringe metropolitan counties (−100.0%; benchmark achieved in both county categories in 2019). In all county categories, preventable premature deaths from unintentional injury increased, with the largest increases occurring in large central metropolitan (+147.5%) and large fringe metropolitan (+97.5%) counties. Preventable premature deaths from CLRD decreased most in large central metropolitan counties where the benchmark was achieved in 2019 and increased slightly in noncore counties (+0.8%). In all county categories, preventable premature deaths from stroke decreased from 2010 to 2013, remained constant from 2013 to 2019, and then increased in 2020 at the start of the COVID-19 pandemic. Percentages of preventable premature deaths varied across states by urban-rural county classification during 2010–2022.

**Interpretation:**

During 2010–2022, nonmetropolitan counties had higher percentages of preventable premature deaths from the five leading causes of death than did metropolitan counties nationwide, across public health regions, and in most states. The gap between the most rural and most urban counties for preventable premature deaths increased during 2010–2022 for four causes of death (cancer, heart disease, CLRD, and stroke) and decreased for unintentional injury. Urban and suburban counties (large central metropolitan, large fringe metropolitan, medium metropolitan, and small metropolitan) experienced increases in preventable premature deaths from unintentional injury during 2010–2022, leading to a narrower gap between the already high (approximately 69% in 2022) percentage of preventable premature deaths in noncore and micropolitan counties. Sharp increases in preventable premature deaths from unintentional injury, heart disease, and stroke were observed in 2020, whereas preventable premature deaths from CLRD and cancer continued to decline. CLRD deaths decreased during 2017–2020 but increased in 2022. An increase in the percentage of preventable premature deaths for multiple leading causes of death was observed in 2020 and was likely associated with COVID-19–related conditions that contributed to increased mortality from heart disease and stroke.

**Public Health Action:**

Routine tracking of preventable premature deaths based on urban-rural county classification might enable public health departments to identify and monitor geographic disparities in health outcomes. These disparities might be related to different levels of access to health care, social determinants of health, and other risk factors. Identifying areas with a high prevalence of potentially preventable mortality might be informative for interventions.

## Introduction

Premature deaths, all-cause mortality, and poor health outcomes are greater among residents of rural counties than of urban counties in the United States (*1*). In 2021, the all-cause age-adjusted death rate in the United States was 841.6 per 100,000 population. The gap in all-cause mortality between rural (nonmetropolitan) and urban (metropolitan) areas of the United States continues to widen. In 1999, the death rate in rural areas was 7% higher than in urban areas; by 2019, it was 20% higher (*2*). Describing premature mortality rates from the five leading causes of death (cancer, unintentional injury [e.g., unintentional poisoning including drug overdose, unintentional motor vehicle traffic crash, unintentional drowning, and unintentional fall], heart disease, stroke, and chronic lower respiratory disease [CLRD]) and related rural disparities might help guide public health messaging and interventions.

The risk for premature death is associated with modifiable factors that vary by disease (*3*). Four of the five leading risk factors for premature death are more prevalent in rural areas of the United States: using tobacco, obesity, physical inactivity, and drinking alcohol or drinking in excess (*4*,*5*). Extensive literature on social determinants of health has established the importance of community context in shaping all aspects of health (*6*). Structural factors (e.g., lower socioeconomic status, limited access to health care professionals, and limited job opportunities) increase the risk for premature death among rural residents (*7*).

Multiple factors influence the rural-urban gap in preventable premature deaths. Because each of the five leading causes of death is age related, these conditions are more prevalent in rural areas of the United States where residents typically are older than their urban counterparts. Working-age adults might leave rural areas to seek better economic opportunities elsewhere (*8*), and older persons might be more likely to retire in rural areas (*9*). However, the population’s age structure alone does not explain the disparity in mortality. Instead, differences in social circumstances, socioeconomic characteristics, health-related behaviors, and access to health care services affect mortality and potentially contribute to approximately half of all preventable premature deaths (*10*). County-level disparities in all-cause premature deaths by rurality, race, and ethnicity have been documented (*11*). Data on cause-specific preventable premature deaths from the leading causes of death by rurality, sex, race, and ethnicity are limited, and direct comparisons accounted for by these factors will be reported in subsequent analyses.

Rural public health needs and sociodemographic characteristics of rural populations are changing (*12*). Although the proportion of the U.S. population that lives in rural areas is gradually declining, any rural population growth can be attributed to in-migration, which might require sensitivity to cultural differences (*13*,*14*). With gradual declines in population, the wealth and tax bases of rural counties also are decreasing, resulting in reduced funding for social and health services (*15*).

In this analysis, mortality data were used to estimate the number and percentage of deaths from each of the five leading causes of death that could have been prevented if all states had similarly low death rates. Disparities in premature mortality from the five leading causes of death in rural areas in the United States during 2010–2022 also were estimated. The results of this analysis are intended to serve as a critical resource for policymakers, public health officials, and researchers striving to understand and address the root causes of preventable premature deaths.

## Methods

This analysis used mortality data for U.S. residents from the National Vital Statistics System (https://www.cdc.gov/nchs/index.htm) to calculate preventable premature deaths by urban-rural county classification from the five leading causes of death during 2010–2022 (heart disease, cancer, unintentional injury, CLRD, and stroke). Deaths from COVID-19 were excluded to maintain consistency and facilitate the assessment of trends over time. Data for 2022 are provisional counts from January through June and were annualized for comparability with previous years.

The number of preventable premature deaths for a specific cause (also described as potentially preventable premature or excess deaths) is equal to the difference in the number of observed deaths among persons aged <80 years and the number of deaths expected if the mortality rate in all states were equivalent to the average rate of the three states with the lowest mortality. Rates in the three states define the benchmarks. The benchmark for each cause of death is derived from a unique set of three states.

Rural and urban categories were identified using the National Center for Health Statistics 2013 urban-rural classification scheme for counties (*16*). County of residence of the decedent was used to determine urban-rural county classification. The categories are 1: large central metropolitan (most urban), 2: large fringe metropolitan, 3: medium metropolitan, 4: small metropolitan, 5: micropolitan, and 6: noncore (most rural).

Preventable premature deaths were calculated individually for the two nonmetropolitan categories (micropolitan and noncore) and the four metropolitan categories (large central metropolitan, large fringe metropolitan, medium metropolitan, and small metropolitan) as well as for the broader categories of metropolitan and nonmetropolitan. Analyses were restricted to deaths with an underlying cause of death from the five leading causes of death based on the *International Classification of Diseases, 10th Revision* (ICD-10): heart disease (I00–I09, I11, I13, and I20–I51), cancer (C00–C97), unintentional injury (V01–X59 and Y85–Y86), CLRD (J40–J47), and stroke (I60–I69). The analysis of preventable premature deaths during 2010–2022 was restricted to persons aged <80 years at the time of death. The age restriction is consistent with the average life expectancy for the U.S. population in 2010, which was approximately 79 years (*17*).

Age-specific mortality rates for each of the five leading causes of death were used to derive the number of preventable premature deaths using methods described elsewhere (*18*). Age groupings varied by cause of death. (Most were 10-year age groups; however, the size of the youngest age group ranged from 0 to 9 years for unintentional injury to 0 to 49 years for CLRD and cerebrovascular disease because deaths from those causes are rare among younger persons.) For each age group and cause of death, the death rates of the three states with the lowest rates during 2008–2010 (benchmark states) were averaged to produce benchmark rates (*18*) (https://stacks.cdc.gov/view/cdc/42342). These benchmarks were chosen to represent the lowest death rates achievable by states at the beginning of the study period and did not vary by year to allow for the examination of trends over time. Although using time-varying benchmarks would better account for potential improvements over time in the benchmark rates, time-varying benchmarks also would make temporal and geographic comparisons more difficult. The same benchmarks were applied to both nonmetropolitan and metropolitan counties, and benchmarks were not adjusted for other characteristics that might affect death rates (e.g., race, ethnicity, socioeconomic status, and urbanicity). Deaths attributed to COVID-19 from 2020 through June 2022 were excluded from this study.

The numbers of preventable premature deaths for each cause of death were assumed to follow a Poisson distribution. SEs were calculated using standard formulas that incorporated the variance around both the observed and the expected counts (*18*), and pairwise z-tests were performed to determine whether the differences during 2010–2022 were statistically significant (p<0.05). All differences during 2010–2022 are statistically significant unless otherwise noted. The percentage of preventable premature deaths was calculated by dividing the number of preventable premature deaths by the total observed number of premature deaths.

## Results

**FIGURE 1:**
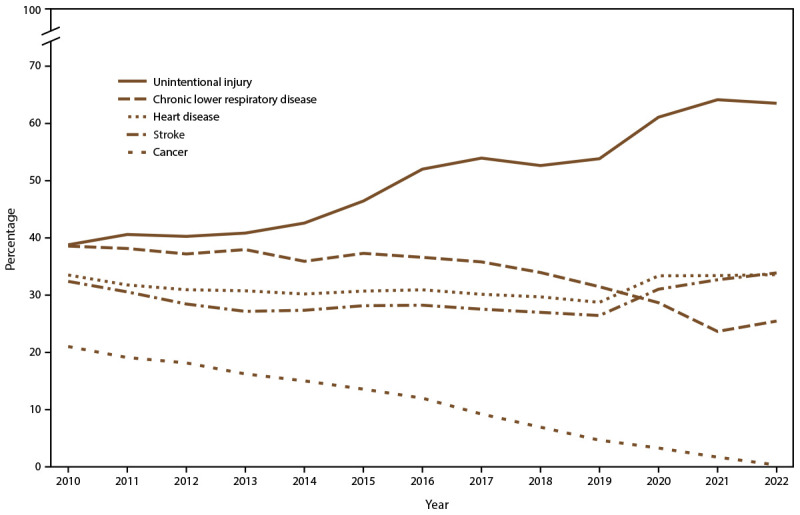
Percentages of preventable premature deaths* among persons aged <80 years from the five leading causes of death, by year — National Vital Statistics System, United States, 2010–2022^†^ * Preventable premature deaths are defined as deaths among persons aged <80 years in excess of the number that would be expected if the death rates for each cause in all states were equivalent to those in the benchmark states (i.e., the three states with the lowest rates). ^†^ Data for 2022 are provisional counts from January through June and were annualized for comparability with previous years.

**FIGURE 2:**
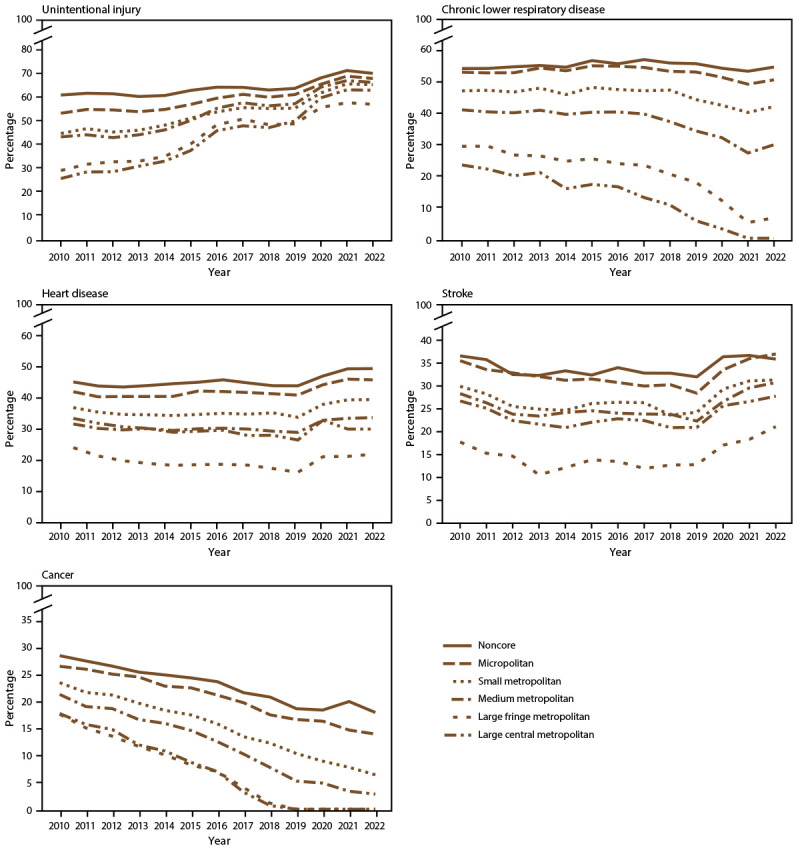
Percentages of preventable premature deaths* among persons aged <80 years from the five leading causes of death, by rural-urban county classification and year — National Vital Statistics System, United States, 2010–2022^†^ * Preventable premature deaths are defined as deaths among persons aged <80 years in excess of the number that would be expected if the death rates for each cause in all states were equivalent to those in the benchmark states (i.e., the three states with the lowest rates). ^†^ Data for 2022 are provisional counts from January through June and were annualized for comparability with previous years.

The percentage of preventable premature deaths from cancer decreased from 2010 through June 2022 (from 21% to 0.3%) (Figure 1). Regardless of urban-rural classification, all county categories experienced decreases (Figure 2). However, the decreases in urban counties were larger than those in rural counties, which widened the rural-urban disparities in preventable premature deaths from cancer (Figure 2) (Table). The percentage of preventable premature deaths from cancer in noncore counties in 2022 (18.1%) was similar to the percentage in large central metropolitan counties in 2010 (17.9%).

**TABLE:** Numbers and percentages of preventable premature deaths* among persons aged <80 years from the five leading causes of death, by rural-urban county classification — National Vital Statistics System, United States, 2010–2022

Cause	2010 No. (%)	2011 No. (%)	2012 No. (%)	2013 No. (%)	2014 No. (%)	2015 No. (%)	2016 No. (%)	2017 No. (%)	2018 No. (%)	2019 No. (%)	2020 No. (%)	2021 No. (%)	2022^†^ No. (%)	Percent change from 2010 to 2022
**Heart disease**														
Large central metropolitan	24,859 (33.5)	23,836 (31.9)	23,173 (30.8)	23,344 (30.3)	22,655 (29.0)	23,660 (29.4)	24,515 (29.7)	23,496 (28.0)	24,158 (28.1)	22,929 (26.6)	31,779 (32.9)	28,410 (30.0)	14,212 (30.1)	−10.2
Large fringe metropolitan	13,945 (24.1)	12,360 (21.4)	11,574 (19.9)	11,358 (19.0)	11,261 (18.4)	11,762 (18.6)	12,159 (18.7)	12,399 (18.5)	11,887 (17.4)	11,184 (16.2)	16,012 (21.2)	16,593 (21.3)	8,635 (22.0)	−8.8
Medium metropolitan	17,781 (31.7)	17,089 (30.3)	17,130 (29.8)	17,992 (30.2)	17,999 (29.6)	18,951 (30.1)	19,583 (30.3)	20,029 (30.1)	19,963 (29.4)	20,148 (29.0)	24,813 (32.9)	26,038 (33.5)	13,143 (33.7)	6.4
Small metropolitan	10,420 (36.9)	10,021 (35.5)	9,924 (34.7)	10,147 (34.7)	10,295 (34.4)	10,686 (34.7)	11,089 (35.1)	11,282 (34.8)	11,822 (35.2)	11,378 (33.8)	13,943 (37.9)	15,089 (39.4)	7,579 (39.5)	7.0
Micropolitan	12,913 (42.0)	12,320 (40.4)	12,585 (40.5)	12,861 (40.5)	13,139 (40.5)	14,326 (42.2)	14,445 (42.1)	14,611 (41.8)	14,686 (41.4)	14,711 (40.9)	17,167 (44.3)	18,723 (46.1)	9,285 (45.8)	9.1
Noncore	11,509 (45.1)	11,111 (43.9)	11,132 (43.5)	11,556 (44.0)	12,015 (44.6)	12,384 (45.1)	12,892 (45.8)	12,644 (44.9)	12,399 (43.9)	12,577 (43.9)	14,482 (47.1)	15,910 (49.4)	7,981 (49.4)	9.5
**Cancer**														
Large central metropolitan	18,754 (17.9)	16,709 (15.8)	15,945 (14.9)	12,844 (12.0)	11,830 (10.9)	9,546 (8.7)	7,693 (7.0)	3,443 (3.2)	736 (0.7)	0 (0)	0 (0)	0 (0)	0 (0)	−100.0
Large fringe metropolitan	16,566 (17.7)	14,208 (15.2)	12,930 (13.6)	11,229 (11.7)	9,870 (10.1)	8,102 (8.3)	6,948 (7.0)	4,000 (4.0)	1,112 (1.1)	0 (0)	0 (0)	0 (0)	0 (0)	−100.0
Medium metropolitan	18,252 (21.4)	16,330 (19.2)	16,337 (18.8)	14,635 (16.7)	14,282 (16.0)	13,232 (14.7)	11,366 (12.6)	9,328 (10.3)	7,057 (7.8)	4,761 (5.3)	4,512 (4.9)	3,117 (3.4)	1,302 (2.8)	−86.9
Small metropolitan	9,606 (23.6)	8,883 (21.8)	8,836 (21.3)	8,263 (19.8)	7,773 (18.4)	7,500 (17.6)	6,798 (15.9)	5,766 (13.5)	5,322 (12.3)	4,497 (10.4)	3,908 (9.0)	3,422 (7.8)	1,383 (6.4)	−72.8
Micropolitan	11,313 (26.7)	11,254 (26.1)	10,912 (25.2)	10,837 (24.7)	10,060 (23.0)	10,050 (22.6)	9,416 (21.3)	8,819 (19.9)	7,758 (17.6)	7,438 (16.7)	7,399 (16.4)	6,619 (14.8)	3,115 (14.0)	−47.4
Noncore	9,806 (28.7)	9,503 (27.7)	9,192 (26.7)	8,831 (25.6)	8,723 (25.1)	8,563 (24.5)	8,321 (23.8)	7,538 (21.7)	7,291 (20.9)	6,456 (18.8)	6,434 (18.5)	7,139 (20.1)	3,130 (18.1)	−37.0
**Unintentional injury^§^**														
Large central metropolitan	5,924 (25.4)	6,941 (28.2)	7,072 (28.2)	8,024 (30.6)	9,021 (32.8)	11,168 (37.3)	15,900 (45.7)	17,580 (47.8)	17,140 (47.0)	19,258 (49.8)	28,868 (59.6)	33,518 (63.0)	16,603 (62.8)	147.5
Large fringe metropolitan	5,765 (28.8)	6,645 (31.5)	7,059 (32.5)	7,229 (32.7)	8,049 (34.8)	10,236 (40.1)	14,422 (48.2)	16,118 (50.6)	14,814 (48.2)	15,219 (48.6)	20,303 (55.5)	22,544 (57.6)	10,953 (56.9)	97.5
Medium metropolitan	9,129 (43.1)	9,589 (43.9)	9,242 (42.8)	9,804 (43.9)	10,808 (46.0)	12,849 (50.1)	15,930 (55.2)	17,774 (57.6)	17,023 (56.2)	17,824 (57.1)	24,187 (64.2)	27,794 (67.0)	13,439 (66.2)	53.7
Small metropolitan	4,342 (44.5)	4,775 (46.6)	4,546 (45.1)	4,715 (45.9)	5,197 (48.0)	5,892 (51.0)	6,587 (53.6)	7,171 (55.5)	7,129 (55.1)	7,213 (55.2)	9,511 (61.7)	11,339 (65.5)	5,595 (65.2)	46.6
Micropolitan	5,865 (53.1)	6,293 (54.7)	6,263 (54.5)	6,107 (53.7)	6,375 (54.7)	6,973 (56.8)	7,786 (59.5)	8,376 (61.1)	8,007 (59.9)	8,418 (61.0)	10,200 (65.4)	12,042 (68.9)	5,755 (67.9)	27.8
Noncore	5,781 (60.9)	5,972 (61.6)	5,924 (61.4)	5,656 (60.2)	5,758 (60.6)	6,304 (62.8)	6,683 (64.2)	6,673 (64.1)	6,379 (63.0)	6,571 (63.6)	8,028 (68.1)	9,289 (71.2)	4,384 (70.0)	15.1
**Chronic lower respiratory disease**														
Large central metropolitan	3,756 (23.4)	3,587 (22.1)	3,250 (20.0)	3,581 (21.0)	2,627 (15.9)	2,986 (17.2)	2,906 (16.5)	2,322 (13.0)	1,902 (10.7)	977 (5.6)	524 (3.0)	0 (0)	0 (0)	−100.0
Large fringe metropolitan	4,546 (29.4)	4,717 (29.5)	4,244 (26.6)	4,357 (26.4)	4,136 (24.6)	4,472 (25.5)	4,237 (23.9)	4,314 (23.4)	3,787 (20.5)	3,305 (17.9)	2,152 (12.1)	855 (5.0)	564 (6.5)	−77.8
Medium metropolitan	6,794 (41.1)	6,815 (40.4)	6,928 (40.1)	7,426 (41.0)	7,275 (39.6)	7,714 (40.3)	7,970 (40.4)	8,104 (39.8)	7,573 (37.3)	6,873 (34.3)	6,432 (32.2)	5,195 (27.3)	2,955 (29.9)	−27.2
Small metropolitan	4,079 (47.1)	4,225 (47.3)	4,243 (46.8)	4,613 (48.1)	4,377 (46.0)	4,928 (48.3)	4,916 (47.6)	5,024 (47.2)	5,244 (47.4)	4,777 (44.4)	4,564 (42.5)	4,230 (40.3)	2,281 (42.1)	−10.7
Micropolitan	5,203 (53.1)	5,284 (52.9)	5,413 (53.0)	5,906 (54.5)	5,843 (53.6)	6,355 (55.2)	6,441 (55.0)	6,538 (54.6)	6,394 (53.4)	6,491 (53.2)	6,210 (51.5)	5,767 (49.3)	3,050 (50.7)	−4.6
Noncore	4,342 (54.3)	4,434 (54.3)	4,621 (54.9)	4,807 (55.3)	4,801 (54.7)	5,307 (56.8)	5,153 (55.8)	5,586 (57.1)	5,469 (56.1)	5,524 (55.8)	5,307 (54.4)	5,116 (53.4)	2,699 (54.8)	0.8
**Stroke**														
Large central metropolitan	4,465 (31.7)	4,263 (30.1)	3,838 (27.5)	3,806 (26.7)	3,779 (25.9)	4,132 (27.1)	4,391 (27.9)	4,506 (27.4)	4,280 (25.9)	4,412 (26.0)	5,713 (30.7)	6,082 (31.6)	3,209 (32.8)	3.4
Large fringe metropolitan	2,494 (22.7)	2,227 (20.3)	2,209 (19.7)	1,732 (15.6)	2,009 (17.1)	2,333 (18.9)	2,345 (18.5)	2,209 (17.0)	2,406 (17.7)	2,494 (17.8)	3,369 (22.1)	3,720 (23.3)	2,160 (26.1)	14.8
Medium metropolitan	3,762 (33.3)	3,527 (31.3)	3,220 (28.9)	3,258 (28.4)	3,492 (29.2)	3,665 (29.6)	3,667 (29.1)	3,784 (28.9)	3,905 (28.8)	3,736 (27.3)	4,732 (31.6)	5,527 (34.6)	2,904 (35.7)	7.2
Small metropolitan	1,886 (34.9)	1,795 (33.3)	1,623 (30.5)	1,625 (29.9)	1,651 (29.7)	1,817 (31.2)	1,882 (31.4)	1,941 (31.4)	1,760 (28.6)	1,866 (29.3)	2,422 (34.3)	2,667 (36.1)	1,345 (36.3)	4.0
Micropolitan	2,393 (40.5)	2,255 (38.6)	2,229 (37.9)	2,211 (37.1)	2,183 (36.3)	2,251 (36.5)	2,212 (35.8)	2,201 (35.0)	2,287 (35.3)	2,155 (33.4)	2,751 (38.6)	3,087 (41.0)	1,610 (42.0)	3.6
Noncore	1,970 (41.6)	1,939 (40.8)	1,723 (37.5)	1,745 (37.3)	1,854 (38.3)	1,808 (37.4)	1,956 (39.0)	1,906 (37.8)	1,944 (37.8)	1,914 (37.0)	2,341 (41.4)	2,371 (41.7)	1,147 (40.9)	−1.7

* Preventable premature deaths are defined as deaths among persons aged <80 years in excess of the number that would be expected if the death rates for each cause in all states were equivalent to those in the benchmark states (i.e., the three states with the lowest rates). Estimates of potentially excess deaths that were negative were set to zero. These negative excess estimates occurred in cases where deaths were fewer than expected (i.e., mortality was lower than benchmark rates).

^†^ Data for 2022 are provisional counts from January through June and were annualized for comparability with previous years.

^§^ Includes unintentional poisoning (e.g., drug overdose), unintentional motor vehicle traffic crash, unintentional drowning, and unintentional fall (https://www.cdc.gov/injury/wisqars/animated-leading-causes.html).

The percentage of preventable premature deaths from heart disease decreased from 2010 through 2019 (from 33.5% to 28.8%), followed by a steep increase to 33.6% from 2020 through June 2022 (Figure 1). Increases from 2020 through June 2022 occurred across all rural-urban categories except for large central metropolitan counties, which experienced a decrease from 32.9% in 2020 to 30.1% in 2021 (Figure 2) (Table). Rural counties had the highest percentage of preventable premature deaths from heart disease in 2022 (45.8% in micropolitan counties and 49.4% in noncore counties) (Figure 2) (Table). Most states experienced an increase in preventable early deaths from heart disease and stroke (96% and 88% of states, respectively) from 2019 through June 2022 (Supplementary Table, https://stacks.cdc.gov/view/cdc/147842).

The percentage of preventable premature deaths from unintentional injury increased from 2010 to 2019 (from 38.8% to 53.8%), followed by a steep increase from 2019 to 2021 and a slight decrease through June 2022 to 63.5% (Figure 1). Increases in preventable premature deaths from unintentional injury during 2010–2022 were statistically significant for all metropolitan categories except micropolitan. Rural percentages were higher than in urban areas, but the gap narrowed (Figure 2). The percentages increased in all states except Wyoming, but the increase varied widely at the state level (Supplementary Table, https://stacks.cdc.gov/view/cdc/147842).

The percentage of preventable premature deaths from CLRD decreased from 2010 through 2022 (from 38.6% to 25.5%) (Figure 1). The percentage of preventable premature deaths varied widely when stratified by rural-urban county category, but all county categories except for noncore counties experienced decreases. Rural-urban disparities widened when large central metropolitan percentages decreased from 23.4% in 2010 to 0% in 2022, whereas the rural percentages hovered between 50.7% and 54.8% in 2022 (Figure 2) (Table).

The percentage of preventable premature deaths from stroke decreased slightly from 2010 through 2019 (32.4% to 26.4%), followed by an increase to 33.9% through June 2022 (Figure 1). Each rural-urban category experienced steep increases from 2019 to June 2022, except for noncore counties that experienced a slight decrease from 2021 to June 2022; rural counties had the highest percentages from January to June 2022 (42.0% in micropolitan counties and 40.9% in noncore counties) (Figure 2) (Table). The highest percentages of preventable premature deaths from stroke in 2022 were in southern states (Supplementary Table, https://stacks.cdc.gov/view/cdc/147842).

## Discussion

Rural residents, particularly those in noncore counties, experienced high percentages of preventable premature deaths during the study period. The rural-urban disparities in premature deaths varied by cause of death. However, disparities were not limited to place of residence. Disparities in all-cause premature deaths also were associated with other demographic factors (e.g., sex, race, and ethnicity) (*11*). For example, the highest rates of premature deaths were observed in rural counties where a majority of the population was Black, African American, American Indian, or Alaska Native (*11*). To address disparities in preventable premature deaths across rural and urban counties, data on disparities in cause-specific premature deaths from the five leading causes by rural-urban county category, race, and ethnicity are needed to inform interventions and health care policies for specific racial and ethnic groups. A follow-up of this analysis stratified by race and ethnicity will be published in subsequent reports, further contributing evidence to guide existing and new programs and policies.

### Cancer

Overall, the decrease in preventable premature deaths from cancer was substantial and was greatest in urban counties where access to preventive services, treatment, survivor care, and specialty care is much higher than in rural counties (*19*). Large central metropolitan and fringe metropolitan areas achieved the benchmark rates in 2019. This is consistent with overall declines in cancer mortality, which decreased 27% between 2001 and 2020 (*20*). The decrease in preventable premature deaths likely reflects multiple factors. Increases in recommended screening for the leading causes of deaths from cancer (e.g., lung, colon, cervical, and female breast) have led to earlier detection, when treatment is more effective, and prevention by detecting cellular changes before they turn into cancer, as in the case of colorectal cancer (*21*). Increases in vaccination rates for cancer-causing viruses and decreases in prevalence of risk factors (e.g., combustible tobacco use) also have driven cancer mortality downward (*22*). Access to these cancer prevention and early detection strategies was increased with the expansion of Medicaid (*23*). New cancer treatments and therapies, specifically for lung cancer and melanoma, also have led to longer survival for those with a cancer diagnosis (*24*). CDC conducted a demonstration project on how to best provide care for persons living in rural areas who had cancer diagnosed (*25*). Although cancer is categorized as a single disease group in this analysis, each cancer site has different risk factors, has varying treatment methods, and can manifest itself in different ways among groups by sex, age, race, and ethnicity. Preventable premature deaths might vary depending on the cancer site and might not have decreased for cancers with increasing prevalence of risk factors (e.g., obesity), no recommended screening modalities, or therapies that have not changed. Lung cancer, the leading cause of cancer mortality, accounted for 23% of all cancer deaths in 2020 (*20*). Geographic differences in combustible tobacco use and use of lung cancer screening likely partially drive differences in lung cancer mortality. Access to lung cancer screening facilities is more limited in rural counties than in urban counties (*26*). Despite overall reductions in preventable premature deaths from cancer, premature deaths surpass the national average in micropolitan and noncore counties, highlighting the need in rural areas to reduce cancer-related premature deaths. Because more urban areas surpassed the 2010 benchmarks for cancer death rates in 2019, future updates to the cancer-specific benchmarks using more recent years of data might better reflect the lowest achievable death rates.

### Unintentional Injury

The worsening and expanding drug overdose epidemic, increases in motor vehicle traffic fatalities, and falls drive the growth in preventable premature deaths from unintentional injury (*27*). Narrowing rural-urban disparities in the percentage of preventable premature deaths from unintentional injury were driven by worsening rates of preventable mortality in more urban areas, with the percentage more than doubling in large central metropolitan areas over the study period. For drug overdoses, access to medications for opioid use disorder continues to be more limited in rural counties, as evidenced by low buprenorphine dispensing rates and reduced treatment capacity (*28*). For motor vehicle traffic crashes, rural residents have an increased risk for death and are less likely than urban residents to wear seat belts (*29*). Evidence-based interventions reduce rural-urban disparities in seat belt use and motor vehicle death rates (*30*). Many fall risk factors are modifiable, implying that many falls can be prevented (*31*).

### Heart Disease and Stroke

Disparities in preventable premature deaths from heart disease and stroke between rural and urban areas existed across the study period. These gaps increased from 2019 to June 2022, except in large central metropolitan counties where a decrease of three percentage points was observed from 2020 to 2021. Increases in preventable premature deaths from heart disease and stroke in 2020 and 2021 were likely associated with COVID-19–related conditions that contributed to risk-associated increased mortality from heart disease and stroke (*32*). Increases in systolic and diastolic blood pressure, a leading risk factor for heart disease and stroke, were observed among all age groups when comparing 2020 with 2019 (*33*). Inequities in control of hypertension (i.e., systolic blood pressure values of ≥130 mm Hg, diastolic blood pressure of >80 mm Hg, or both) were observed during the COVID-19 pandemic and are related to insufficient health care access, medication adherence, and monitoring (*34*). Patients might have delayed or avoided seeking emergency care when experiencing a life-threatening event during the height of the COVID-19 pandemic (*35*). Emergency department visits for heart attack and stroke decreased by 20% during the weeks after the declaration of COVID-19 as a national emergency on March 13, 2020, and hospital admissions for heart attack and stroke decreased during the pandemic (*35*). In addition, COVID-19 was associated with an increased risk for stroke and heart disease (*36*,*37*).

### Chronic Lower Respiratory Disease

Despite the overall decrease during 2010–2020 (because of decreases observed in larger urban areas), the percentage of preventable premature deaths from CLRD was relatively stable in medium and small urban counties and rural counties during 2010–2015. During 2010–2022, the sharpest decline in preventable premature death from CLRD in urban areas occurred from 2019 through 2021 and could be the result of deaths from COVID-19 that otherwise would have been attributable to CLRD. Persons with CLRD (e.g., chronic obstructive pulmonary disease) are at increased risk for death from COVID-19 (*38*).

## Limitations

The findings in this report are subject to at least six limitations. First, applying benchmarks (e.g., the three states with the lowest rates) to all urban-rural county categories facilitates comparisons but might not represent the lowest death rates achievable by certain subgroups. For example, large metropolitan areas met the benchmarks for cancer in 2019 and, therefore, had negative estimates of preventable premature deaths during 2019–2022. In these instances, negative estimates were truncated at zero to indicate that the 2010 benchmarks had been achieved. Using urban-rural–specific benchmarks would likely result in larger numbers of preventable premature deaths in certain categories and larger rural-urban disparities in certain cases. However, using benchmarks specific to state, age, cause, and subgroup (e.g., urban-rural, sex, race, and ethnicity) also could lead to less stable estimates because of smaller numbers of deaths when stratifying by multiple demographic and geographic dimensions. Second, the differences cannot be attributed solely to population size and geographic location because risk factors do not occur randomly in populations and are related to well-known social, demographic, environmental, economic, and geographic attributes of the neighborhoods in which persons live and work (*39*). Third, estimates of preventable premature deaths using historical benchmarks (e.g., 2008–2010) might not reflect improvements in mortality that could have occurred in a later year. For example, the 2010 benchmarks for cancer are higher than if benchmarks were based on 2022 data because of decreases in cancer deaths during 2010–2022. Fourth, the numbers of preventable premature deaths by cause are not necessarily independent, and the numbers of potentially excess deaths from the five causes cannot be combined to generate a total. For example, the number of preventable premature deaths from cancer might be lower because persons with cancer died from another cause (e.g., heart disease). Fifth, deaths from certain causes might have increased partly because of COVID-19 and the pandemic. Specifically, misclassification of deaths from COVID-19 that were attributed to other causes (because of lack of testing or reporting on the death certificate) could have contributed to a greater number of deaths across other causes and by rural and urban areas. In addition, certain causes of death might have increased because of indirect pandemic-related effects (e.g., reduced access to emergency care or life-saving treatments). Finally, data for 2022 were based on provisional data from the first half of the year, and results might differ when the final data for the full year are available.

## Future Directions

The findings in this report demonstrate the value of analyzing preventable premature deaths according to the six National Center for Health Statistics 2013 urban-rural county classifications. Reporting trends in preventable premature deaths over a 12-year period highlights differences over time and might aid in understanding underlying structural, environmental, and social risk factors. Because of increasing percentages of preventable premature deaths in recent years for specific causes of death and certain demographic groups, these data might augment traditional rate comparisons and help guide focused public health interventions. Comparing the findings in this report with data from tools such as the CDC Interactive Atlas of Heart Disease and Stroke (https://www.cdc.gov/dhdsp/maps/atlas/index.htm) might help identify social determinants of health, health care infrastructure, and public policies that could be related to increases or decreases in preventable premature deaths in specific nonmetropolitan areas. Detailed community-based evaluations might clarify how various risk factors and social determinants of health relate to premature mortality and related rural-urban disparities. In addition, other methods for developing benchmark rates might be helpful, including using benchmarks based on the nonmetropolitan areas with the lowest death rates, or updating benchmarks based on more recent data, especially for causes of death (e.g., cancer) that have decreased substantially over time. In addition, more detailed analyses by race, ethnicity, and age, along with examining preventable deaths among persons aged >80 years, and preventable premature deaths from other causes (especially causes that are more prevalent in rural counties) might be informative.

## Conclusion

Defining preventable premature deaths within the context of the five leading causes of death does not capture the full spectrum of preventable mortality. The degree to which these deaths could be prevented is related to various factors, including distinct prevention strategies, varying risk factors, and the availability of effective interventions, all of which vary by cause. Not all premature deaths are equally preventable among the leading causes or even within each specific leading cause category, as exemplified by certain types of cancer (*40*).

Routine tracking of preventable premature deaths by urban-rural county classification might facilitate the identification of areas with high prevalence of preventable premature mortality along with related geographic and urban-rural disparities in health outcomes. These disparities might be related to different levels of access to health care, social determinants of health, and other risk factors. Findings might help guide more focused interventions to reduce premature death from the five leading causes of death and reduce disparities by rural-urban residence and geographic region.
